# Acquisition and Development of Verb/Predicate Chaining in Hebrew

**DOI:** 10.3389/fpsyg.2019.02958

**Published:** 2020-01-22

**Authors:** Ruth Berman, Lyle Lustigman

**Affiliations:** ^1^Department of Linguistics, Tel Aviv University, Tel Aviv, Israel; ^2^Department of Communicative Disorders and Sciences, San José State University, San Jose, CA, United States

**Keywords:** caretaker input, discourse connectivity, extended predicates, Hebrew, infinitives, monoclausal constructions, predicate-chaining, verb-chaining

## Abstract

The study considers development and use of verb/predicate chaining constructions by Hebrew speakers from early childhood to adolescence, based on analysis of authentic conversational and narrative corpora. Three types of constructions are analyzed, ordered hierarchically by degree of cohesivity and obligatoriness of chaining: (1) monoclausal complex predicates (the “extended predicates” of traditional Hebrew grammars); (2) coreferential interclausal predicate chaining; and (3) discursively motivated topic chaining. Relevant typological features of Modern Hebrew are reviewed as accounting for the absence of canonical clause chaining in the language (the paucity of non-finite constructions in everyday usage, absence of an uninflected basic form of verbs, lack of auxiliary verbs, and monolexemic verb-internal complexity). Monoclausal verb chaining emerges early in the speech of toddlers in interaction with their caretakers, whereas predicate chaining by coordination across clauses occurs only later, and chunking of such constructions at the service of discourse connectivity is found only from school-age. Non-finite subordination emerges as an advanced form of clause combining, in contrast to straightforward subordination with the multifunctional subordinator *še* ‘that’. Two main conclusions follow from the study: First, the innovative hierarchy defined here for different degrees of verb/predicate linkage mirrors developmental phases in child language; and, second, monoclausal chains of finite verbs or verbal operators followed by infinitival complements are grammatically obligatory, and are common from an early age, whereas bi- and multi-clausal predicate chaining represents an optional rhetorical choice on the part of a given speaker–writer in a particular communicative context.

## 1 Introduction

The study considers acquisition and development of verb and predicate chaining in Modern Hebrew (henceforth MH). Concern is with developing strategies in the formation of two types of constructions: verb chaining in monoclausal complex predicates and predicate chaining in bi- and multi-clause constructions. The goal of the paper is to demonstrate how use of these constructions changes from early pre-school age to adolescence and adulthood in light of general principles underlying the authors’ conception of language acquisition in general. First, development in knowledge and use of complex predicates and clause linkage in Hebrew is analyzed in relation to their functions in discourse rather than as isolated grammatical constructions (Berman, 2016a). Second, development is interpreted in light of a phase-based view of development of knowledge of language and other cognitive domains (Karmiloff-Smith, 1986, 1992), as applied to Hebrew morpho-syntax for early child language (Berman, 1986; Lustigman, 2016a) and later language development (Berman, 2004). In this perspective, analogous developmental processes are recurrent across different domains at different times in a child’s linguistic history, in contrast to the over-arching, domain-general Piagetian view of “stage”. In the case in point here, this means that acquiring knowledge and use of what is labeled below as verb/predicate chaining in Hebrew proceeds from initial pre-grammatical emergence, via piecemeal structure-based knowledge, to integrated, discourse-appropriate mastery at periods which may differ from the same children’s command of, say, inflectional morphology or syntactic subordination in Hebrew. Consequently, even though basic grammatical properties of the target language may be in place by 3 or 5 years of age, language development reflects a lengthy route into adolescence and beyond (Tolchinsky, 2004; Nippold, 2007). A further factor impinging on development, one at the core of the present volume, is the early and profound impact of the typology of the ambient language on the process of acquisition (Choi and Bowerman, 1991; Slobin, 1997; Berman, 2016b).

The language of concern here is the “General Israeli” sociolect (Blanc, 1968; Henkin, 2020) of Hebrew, a Semitic language that was formerly mainly a written means of religious and liturgical study in the Jewish diaspora. Revived since the late 1900s as a means of everyday spoken intercourse, MH serves today as the first language of fourth and fifth generations of children born in Israel and as the primary language of the majority of citizens and residents of the country (Grossman and Reshef, 2020).

The paper is organized as follows: We first outline typological properties of MH relevant to the topic of verb/predicate chaining (§2), then provide details of the analytical framework applied in the study, including predictions based on this analysis (§3), proceeding to description of the database (§4), results of analysis (§5), and a concluding discussion (§6).

## 2 Relevant Features of Mh Morpho-Syntax

The properties of MH surveyed under this heading concern features of the language that mitigate against it being a canonically “clause chaining” language. That is, non-finite clauses in Hebrew are either coordinated with or subordinate to a finite clause, but the language lacks a third category of what is termed “co-subordination” or “asymmetric coordination” (see, further §3 below). Relevant features of MH noted here include: its lack of simplex verb forms (§2.1), verb-internal lexemic complexity (§2.2); lack of auxiliaries and light verbs (§2.3); paucity of non-finite constructions (§2.4); and the asymmetry of person inflection on verbs (§2.5). These for the most part are typologically shared with other Semitic languages (Goldenberger, 2013), while at the same time reflecting current developments in MH usage (Berman, 2020).

### 2.1 Lack of “Basic” Verb Forms

Hebrew verb forms encode a great deal of information verb-internally by means of rich inflectional and derivational morphology. Importantly, the language lacks simplex, uninflected verb forms corresponding to, say, English, *eat, dance, think, enjoy* (Berman, 1977; Lustigman, 2012). As a result, from early on children, like speakers in general, must select some inflected form of any verb they choose to use.

A favored “pre-grammatical” alternative selected by Hebrew-acquiring toddlers when they first start using verbs – at the phase of “emergence,” lasting for one to several months – takes the form of skeletal “bare verbs,” or inflectionally unmarked stems (Berman and Armon-Lotem, 1996; Lustigman, 2013). For example a 2-year-old responds to her mother’s suggestion *ulay* ***te-cayr-i*** *li lecan* ‘Maybe FUT.2-draw-SG.F to.me clown = maybe you’ll draw a clown for me’ by saying *ken*, ***bó-i cayer*** *lecan ‘*Yes, come:IMP-SG.F draw:STEM clown = Yes, come draw a clown^1^’. The child’s utterance is ungrammatical since she uses a juvenile, uninflected “bare verb” stem in the form *cayer* ‘draw’. This child-generated truncated verb form is opaque in isolation, since it could be interpreted as standing for any unmarked irrealis form of the verb – Infinitive *le-cayer* ‘to draw,’ masculine singular Imperative (hence unmarked) Imperative *cayer* ‘draw!’, or Future 1st person plural *ne-cayer* ‘we’ll = let’s draw’. Only the context, rather than the verb form by itself, makes it clear that the child meant the latter, in response to her mother’s suggestion that she draw a clown^2^.

This initial, short-lived but robust phase in early verb acquisition is followed by partial use of inflected verbs, typically in one of the two most neutral verb forms in the language: (i) invariable *infinitives* marked only be prefixal *lV-* (e.g., *li-gmor* ‘to-finish’ in the sense of imperative ‘stop!, finish! – compared with more highly inflected forms of the same verb lexeme like *gamár-ti* ‘finish.PAST-1SG = I (‘ve) finished,’ *ni-gmor* ‘FUT.1PL-finish = we’ll finish’; or (ii) present-tense *benoni* ‘intermediate’ form verbs that are marked for gender (e.g., *roce/roca* ‘want:M/F’ depending on whether the speaker is a boy or girl) and number (*ani roce* ‘I want’/*hem rocim* ‘they want’) but not for person (Lustigman, 2013). By age 3–4 years, typically -developing children have overall command of inflectional distinctions for number (singular/plural), gender (feminine/masculine/feminine), person (1st, 2nd, 3rd); tense (present, past, future); and mood (declarative, imperative) (Berman, 1986).

### 2.2 Verb-Internal Monolexemic Complexity

In addition to the inflections noted in §2.1, all (although not only) verbs in MH are constructed by means of the “root-and-pattern” morphology of Semitic languages (Amberber, 2010; Goldenberger, 2013, pp. 115–118). In Hebrew, these take the form of the *binyan* ‘building = conjugation, construction’ system of 7 verb patterns or prosodic templates, in the form of affixal stems combined with abstract consonantal roots (e.g., Berman, 1993; Ravid et al., 2016). As a result, verb-derivation in MH involves valence-changing alternations that might be expressed analytically in other languages. These includes contrasts of voice – active, passive (both syntactic and adjectival), and middle – which are expressed morphologically, by changes in *binyan.* For example, the abstract historical root *p-t-h* is the basis, in five different *binyan* forms, for the following verb lexemes: *patax* ‘open.TR,’ *niftax* ‘open.INTR = be/get opened,’ *pitéax* ‘develop.TR,’ *putax* ‘be-developed:PASS,’ *hitpatéax* ‘get-developed = develop:INTR; or, from the root *h-p-k*: *hafax* ‘turn, change.TR’/*nehefax* ‘be changed, become,’ *hithapex* ‘turn upside down.INTR^3^’.

The derivational system of *binyan* verb patterns thus encodes several categories that may be expressed compositionally in other languages. For example, causatives that can be expressed with verbs like *make, faire, hacer* in European languages are typically formed by means of a morphologically derived form of a more basic verb or adjective in the *hif’il* pattern (e.g., *le-hardim* ‘make-go-to sleep’ = ‘put to sleep’ from the root *r-d-m*, *le-haaciv* ‘make unhappy’ from the root *?-c-b*). Relatedly, use of intransitive, middle-voice morphology serves in place of inchoative ‘helping verbs’ like *get*, *become, turn* (e.g., *le-hitkonen* ‘get ready,’ *le-hitbayeš* ‘be ashamed,’ or *le-hizaher* ‘take care, be careful,’ *le-hitaka* ‘get stuck’). While sometimes included in the category of “complex predicates” (Alsina et al., 1997, pp. 1–47), particularly those analyzed as “merger constructions” (Baker and Harvey, 2010), these lie outside the present analysis, since the moment speakers use a verb in Hebrew, it must necessarily be made up of both a root and pattern combination, as shown from the very first verbs used by Hebrew-speaking toddlers noted in the preceding section (and see, too, Lustigman, 2012, 2016a). Since consonantal roots are unpronounceable, non-linear elements, and all verbs must have an associated morphological pattern, root plus affixal pattern complexes (phonologically constituting prosodic templates) represent unitary verb *lexemes* in the language.

### 2.3 Lack of Auxiliary and Light Verbs

A feature of MH that mitigates against clause-internal verb chaining is its almost entire lack of auxiliary verbs corresponding to, say, *be, have, do* in Standard Average European (SAE). For present purposes, auxiliaries are narrowly defined as a category of paradigmatically related closed class items that serve for grammatical expression of Tense, Mood, and/or Aspect, as well as Voice (Berman, 1980). A single exception in MH is the multi-functional construction consisting of past tense ‘be’ followed by a *benoni* ‘intermediate’ form participle (see §2.4 below). This serves in current usage to express both habitual past tense as in (1) and ‘unreal’ conditional mood as in (2), where a square bracket] indicates clause boundary and verbs are in bold. However, as shown below, the contrast between the complex *was/were* + *Participle* extended form and the simplex inflected form of the verb to express past tense is optional, and stylistically rather than grammatically required (Berman, 2001).


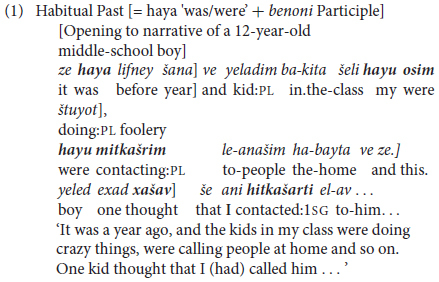


In MH, the habitual past often, though not obligatorily, occurs in the background setting of these personal experience accounts. It is generally optional, alternating with simple past in both such contexts and in general reference to past time in Hebrew (Berman, 2001). In contrast, conditional clauses like the constructed example in (2) require use of auxiliary *haya* ‘be.PST + *benoni* participial construction.


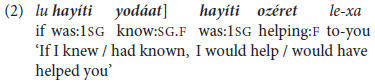


Together with a general lack of auxiliary verbs in favor of monolexemic root-plus-pattern verb formation (§2.2), Hebrew makes relatively infrequent use of light verbs, in contrast to a Semitic language like Amharic, which has complex verbs such as those meaning ‘say’ or ‘do’ along with “morphological encoding of transitivity through the use of various derivational prefixes” (Amberber, 2010). In current Hebrew, certainly in more normative usage, monolexemic verbs are generally preferred to lexically complex constructions. Compare, with verbs in four different *binyan* patterns, *le-hitkaléàx* ‘to-shower (oneself)’ rather than *la-asot mikláxat* ‘to-do, make (a) shower,’ *le-harcot* ‘to-lecture’ rather than *la-tet harcaa* ‘to-give (a) lecture,’ *li-ršom* ‘to-note (down)’ rather than *la-káxat rešimot* ‘to-take notes,’ *le-tayel* ‘to-travel’ rather than *la-cet le-tiyul* ‘go-for (a) trip’.

### 2.4 Non-finite Verbs

Modern Hebrew lacks several of the constructions dealt with in the present volume, including (i) monoclausal *serial verbs*, characterized by Aikhenvald (2018) as monoclausal sequences of verbs that co-occur without an overt marker of coordination, subordination, or syntactic dependency of any sort; and (ii) inter-clausal *converbs*, where a non-finite verb form in the first clause is followed by a finite verb form in the second (Haspelmath and König(eds), 1995; Bickel, 1998). Rather, MH relies extensively for verb and predicate chaining, on the syntactically pervasive and multifunctional **infinitives** corresponding to forms with English ‘to’ or Romance suffixes like *-er, -ir, -ar –* as detailed in Section “Database” below.

Hebrew has two other types of non-finite verb forms defined, following Givón (1990, Chapter 19) as verbs that cannot be used in an independent clause. They thus in some ways correspond to converbs, since they also “take their temporal specification from the tense of the main clause” (Aksu-Koç, 1994, p. 346), but both are obligatory inflected. These are *Gerunds*, as in (3a) and *benoni* ‘Intermediate’ *Participles*, as in (4a), with each having different syntactic functions: Gerunds occur in adverbial clauses of attendant circumstances, and Participles in “small clauses” as complements of verbs of perception. Both function as dependent clauses, and correspond to finite subordinate clauses like those in the constructed examples in (3b) and (4b).


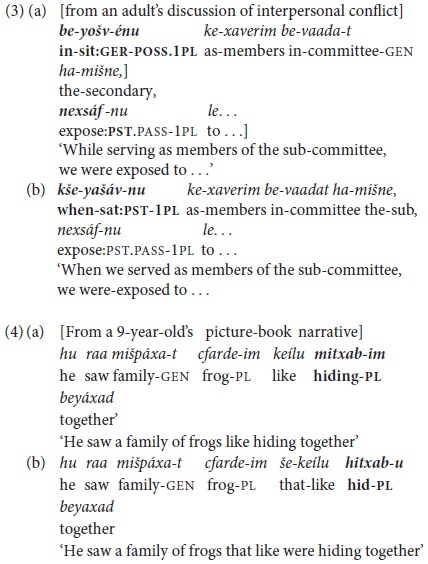


The non-finite gerundives in (3a) and (4a) differ from infinitives since they are obligatorily inflected for agreement of person and/or number, and they can, as shown by the constructed versions in (3b) and (4b), be paraphrased by a finite clause subordinated by a conjunction such as *kše-* ‘like that = when, while’ or *še-* ‘that’. That is, unlike infinitives, they serve only in inter-clausal, not in mono-clausal constructions. Besides, these two major non-finite forms of classical Hebrew – Gerundive *šem ha-póal* ‘verbal noun’ and Participial *benoni* ‘intermediate’ – are today largely confined to formal, elevated or literary usage (Dubnov, 2015; Berman, 2018c). And indeed, across our spoken corpora, from early childhood to graduate student adult usage (§4), non-finite Gerunds and Participles were few and far between – the former largely replaced by finite subordinate clauses as in (3b), the latter by Infinitives. Since neither construction is relevant to the topic of predicate chaining, they are not further considered here. They do, however, highlight (i) the paucity of non-finite verb constructions in MH in contrast to its classical antecedents and (ii) the key role played by infinitives in verb and predicate chaining, as detailed in Section “Framework of Analysis” below.

### 2.5 Asymmetry of Person-Marking

Modern Hebrew can be characterized as “a partial Pro-drop language” due to the asymmetry of where inflectional marking of Person is optional or obligatory on verbs marked for Tense. Thus, verbs are inflected for person in past tense (by suffixes) and in future tense (by prefixes) in 1st and 2nd but not 3rd person, and not on verbs in the *benoni* present tense (Berman, 1990). For example, overt pronouns like *ani* ‘I,’ *atem* ‘you.PL’ are optional in 1st or 2nd person clauses in past or future tense (e.g., *ani nahág-****ti*** *lvad* ‘I drive:PST-1SG alone’ and *nahág-****ti*** *lvad* ‘drive:PST-1SG alone’ both mean ‘I drove alone’; while *atem* ***ti-****nhag-u lvad ‘*you:PL-drive:FUT-PL alone’ and ***ti-****nhag-u lvad* both mean ‘you’ll drive alone’). Analyses show that overt subject pronouns with person-marked verbs are superfluous except in specific discourse contexts (Ariel, 2000; Polak-Yitzhaki, 2006). In contrast, clauses with verbs in present tense or in 3rd person, which are inflectionally marked for number and gender but not for person, require an explicit pronoun as subject. Compare present tense ***ani*** *noheg/et lvad* ‘**I** drive:M/F alone,’ ***at*** *nohéget lvad* ‘**you:SG.F** drive alone,’ ***ata*** *noheg lvad* ‘**you:SG.M** drive alone’; and 3rd person ***hu*** *nahag/****hem*** *nahagu lvad* ‘**he/they** drove alone’ – where the overt subject is obligatory in isolated clauses. As noted further below, this alternation of optional versus obligatory subject pronouns plays an important role in inter-clausal coordination.

In sum, the features of MH usage surveyed above – obligatory verb-internal inflectional and derivational complexity (§2.1 and §2.2), paucity of auxiliaries and light verbs (§2.3) and of non-finite verbs (§2.4), and the partial requirement of overt subject pronouns in isolated clauses (§2.5) – combine with its lack of monoclausal serial verbs and of inter-clausal converbs to explain why Hebrew fails to constitute a canonically “clause chaining” language. As a result, the coding categories delineated in the next section for MH differ from those of more typical clause chaining languages including ones characterized for child language in this volume (for example, Choi on Korean, Ogel-Balaban and Aksu-Koç on Turkish) and see, too, Sarvasy (2019) on the Papuan language of Nungon.

Against this background, the next section outlines the novel analytical framework adopted for the present study of MH, taking into account different levels of structural complexity in specifying coding categories for verb and predicate linkage.

## 3 Framework of Analysis

The basic unit of analysis, in keeping with the theme of this volume, is the **“clause,”** defined as a “unified predication … that expresses a single situation (activity, event, state)” and which includes finite and non-finite verbs, together with its associated arguments and adjuncts (Berman and Slobin, 1994, pp. 660–662). This unit has been effectively applied across different languages, text types, and age-groups, including the narrative database detailed for Hebrew in Section “Database” below (and see, too, for English and Hebrew, Nir-Sagiv, 2008, pp. 48–54). And it accords well with Aikhenvald’s (2018) definition of monoclausal constructions as “describing what is conceptualized as a single event”.

The study differentiates between mono-clausal **multi-verb complex predicates** and inter-clausal (bi- and multi-clausal) **clause combining** constructions. Although, as noted, Hebrew is not a typical clause-chaining language, these two constructions are labeled here, for the sake of consistency with the general topic of this Research Topic, as (mono-clausal) “verb chaining” and (bi- or multi-clausal) “predicate chaining” respectively. Verb chaining is characterized for Hebrew by cases where a tensed verb or verbal operator combines with one or more non-finite forms (most typically infinitive) within a single clause, while predicate chaining involves more than a single clause, typically combined by coordination (similarly to, say, English ‘*pedestrians need to pay attention*] *and not cross at a red light*],’ where] stands for clause boundary). Constructions analyzed in the present study share the following features: (i) Co-reference of the grammatical subject or discursive topic of all the verbs in the chain and (ii) the initial element is marked for tense or mood, and the following non-finite complement(s) inherits the temporal interpretation of this initial element in the main clause. The study thus focuses on non-finite chaining, and excludes from consideration cases of finite subordination (complements, adverbials, and relative clauses) where both the main and dependent clauses are marked for tense. Relevant constructions are defined below along a continuum of three levels of “**depth of dependency,**” ranked by developmental, structural, and/or discursive complexity from (i) the most dependently interwoven monoclausal extended predicates, to (ii) bi- or multi-clausal predicate chaining, followed by the rhetorical option of (iii) discursive topic chaining. This innovative analysis both draws on and departs from earlier studies of two types of syntactic constructions: the mono-clausal “extended predicates” of Hebrew grammars (Berman, 2018b; Lustigman, in press; and see references in footnote 7 below) and “clause combining” complex syntax in Hebrew and English (Berman and Lustigman, 2014; Berman, 2018a).

A key distinction here is that between verb chaining and predicate chaining constructions, as follows. **Verb chaining** (§3.1) applies in monoclausal constructions where a verb, termed the “trigger,” is grammatically finite (marked inflectionally for tense or mood) and semantically encodes modal, aspectual, or evaluative content, and it is complemented by one or more non-finite, typically infinitival, verbs (e.g., *roce/hitxil/ya-adif* ***le-hišaer ba-bayit*** ‘wants/began/will-prefer **to-stay at.the-home,**’ analogously to English *he needs/began to/would prefer to stay at home*). The term “complement” applies in this context to one or more non-finite (infinitive) verbs following the finite trigger in the boundaries of a single clause, rather than in the more usual sense of “sentence complementation” (e.g., Noonan, 2007).^4^ The constructions are analyzed here as constituting the major (in fact, the only) instance of “complex predicates” in MH, in contrast to the varied types of constructions discussed for different languages in Alsina et al. (1997).

**Predicate chaining** (§3.2 and §3.3) involves bi- or multi-clausal constructions, typically combined by coordination. These may be triggered by non-finite verbs in the conjunct clause; for example, compare the present-tense modal verb *crixim* ‘must, have to.PL’ in the first clause followed by the (non-finite) infinitives *la-sim* ‘INF-unput’ in the same clause and *la-xcot* ‘INF-cross’ in the second clause of the following coordinated construction: *holxey-régel* ***crixim la-sim*** *lev]* ***ve*** *lo* ***la-xcot*** *be-or adom* ‘pedestrians need to-pay attention] and not to-cross at a red light,’ where] stands for clause boundary, as in (§3.2.1 and §3.3.1). Alternatively, finite clauses, whether coordinated or subordinated, can also constitute instances of predicate chaining, on condition that they involve same-subject deletion rather than pronominalization of the shared subject in the chain of clauses – e.g., *hu lo sam lev] ve xaca be-or adom* ‘he didn’t pay attention] and crossed at a red light’ (§3.2.2 and §3.3.2).

Constructions defined as manifesting verb or predicate “chaining” and so constituting the coding categories for analysis were selected to meet the following criteria: (i) Co-reference of the grammatical subject or discursive topic of all the verbs in the chain; (ii) the initial element – defined in surface terms as the first verb in a chain – is marked for tense or mood; and (iii) the following verb or verbs in the chain share the same temporal interpretation – whether in the same clause (§3.1), or across different clauses (§3.2 and §3.3). These constraints mean that, with the exception of same-subject deletion in finite coordinated or subordinated clauses (§3.2.2 and §3.3.2), the constructions analyzed below involve non-finite verb forms which, for spoken Hebrew, are largely in the form of Infinitives (§2.4 above).

Results presented in §4 below encompass both mono-clausal contexts, as the primary instance of “complex predicates” in Hebrew (§3.1) as well as in bi-and multi-clausal constructions (§3.2 and §3.3). This motivates the decision to include infinitives, as “deranked” non-finite forms that typically do not take an overt subject (Croft, 2001; Givón, 1990). Besides, concern with constructions including non-finite verbs highlights a largely neglected topic in first language acquisition, with the occasional exception of studies of young preschool children (e.g., Bloom et al., 1984, 1989; Vasilyeva et al., 2008) and for L2 in Döpke (2000) and Akinçi and Jisa (2001). On the other hand, studies of school-age and adolescent students show that non-finite subordination increases markedly from adolescence on, as in Berman’s (2003) analysis of English and Hebrew narratives, Jisa (2000, 2004), studies of French L1 texts, and Kupersmitt’s work comparing temporality in English, Hebrew, and Spanish.

Grammatically, such constructions manifest a tightly bound type of combining, since in them, the subject and tense of the subordinate clause are totally dependent on the main clause (Van Valin and LaPolla, 1997). In discourse-embedded terms, non-finite chaining represents a particularly tightly cohesive type of “syntactic packaging” or “discursive chunking” of texts, due to what Givón (2009) terms their “semantic integration” (and see, too, Cristofaro, 2003, pp. 117–122, cross-linguistic analysis of the functions of non-finiteness).^5^ Relatedly, Popa (2008) emphasizes the important role of what she terms “theme ellipsis” (of verbless and non-finite clauses) in the information structure and overall organization of English texts, while Maiá (2010) defines non-finite clauses as contributing to the “structural compactness” of different types of texts she analyzed in English.

Relevant constructions are defined below along a continuum of three levels of “**depth of dependency,**” ranked by structural, and/or discursive complexity from the most obligatory and inter-dependent monoclausal complex predicates (§3.1) via bi- or multi-clausal predicate chaining (§3.2), and on to the rhetorical option of discursive topic chaining (§3.3). The section concludes with formulation of relevant developmental predictions (§3.4). The study differentiates between mono-clausal **multi-verb complex predicates** and inter-clausal (bi- and multi-clausal) **clause combining** constructions. Although, as will be evident from the description in (§1.2 and §1.3) and analyses in (§4.2 and §4.3), Hebrew is not a typical clause chaining language, these two constructions are labeled here, for the sake of consistency with the general topic of this Research Topic, as (mono-clausal) “verb chaining” and (bi- or multi-clausal) “predicate chaining” respectively. Verb chaining is characterized for Hebrew (§1.1) by cases where a tensed verb or verbal operator combines with one or more non-finite forms (most typically infinitive), while predicate chaining (§1.1–§1.3) involves more than a single clause, typically combined by coordination (similarly to, say, English ‘*pedestrians need to pay attention*] *and not cross at a red light]*,’ where] stands for clause boundary). Constructions analyzed in the present study share the following features: (i) Co-reference of the grammatical subject or discursive topic of all the verbs in the chain and (ii) the initial element is marked for tense or mood, and the following non-finite complement(s) inherits the temporal interpretation of this initial element in the main clause. The study thus focuses on non-finite chaining, and excludes from consideration cases of finite subordination (complements, adverbials, and relative clauses) where both the main and dependent clauses are marked for tense. Relevant constructions are defined below along a continuum of three levels of “**depth of dependency,**” ranked by developmental, structural, and/or discursive complexity from (i) the most dependently interwoven monoclausal extended predicates, to (ii) bi- or multi-clausal predicate chaining, followed by the rhetorical option of (iii) discursive topic chaining. This innovative analysis both draws on and departs from earlier studies of two types of syntactic constructions: the mono-clausal “extended predicates” of Hebrew grammars (Berman, 2018c; Lustigman, in press) and “clause combining” complex syntax in Hebrew and English (Berman and Lustigman, 2014; Berman, 2018a). These are, as noted, labeled respectively (mono-clausal) verb chaining and (bi- or multi-clausal) predicate chaining for the sake of consistency with the type of constructions focused on in other chapters in this Research Topic.

### 3.1 Mono-Clausal Complex Predicates

The first level concerns what Givón (2009, 128–136) refers to as “complex verb phrases”. In Hebrew, these take the form of mono-clausal complex predicate constructions consisting of a tensed verb or operator (semantically modal, aspectual, or evaluative) followed by a single infinitive (§3.1.1) or by two or more infinitives (§3.1.2).

#### 3.1.1 Single Trigger

Two constructions are considered under this heading: a finite verb or verbal operator followed (i) by an infinitive and (ii) directives consisting of an imperative verb meaning ‘come, go’ followed by a verb in the infinitive or inflected for future tense/imperative mood. The first of these constructions, a key facet of Hebrew syntax at different ages, registers, and text-types is illustrated in by utterances produced by a 2-year-old girl in (5a) and a graduate student woman in (5b).


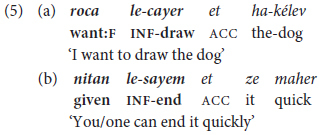


The expressions bolded in (5) illustrate constructions traditionally termed *nasu murxav* literally ‘predicate widened = extended predicate’ in Hebrew grammars (Lustigman, in press). These take the form of tense-marked, finite verbs or verbal operators followed by a non-finite verb, and they constitute **the** means par excellence for elaborating VP constructions in a language lacking in auxiliary verbs and other multi-lexemic means of verb expansion (see §2.3 above). As illustrated in (5), they take the form of (at least) two verbal elements, the first a verb or verbal operator inflected for tense or mood followed by one or more non-finite verbs, typically in the infinitive. Semantically, the initial item, here termed the “trigger,” is generally modal (e.g., *want to, have to, need to, ought to*), aspectual (e.g., in the equivalents of *be going to, start to, go on, stop*), or expresses evaluative *cum* attitudinal content such as *(dis)like, prefer, avoid.^6^* Morphologically, the trigger is not necessarily a canonic verb, but may be part of a defective or suppletive verbal paradigm (e.g., *carix* ‘should, have to,’ *yaxol* ‘can, be able to’), or they may be adjectives [e.g., *muxrax* ‘must, be-bound-to,’ *mutar* ‘allowed to,’ *efšar* ‘(it’s) possible’], or even nouns (*xaval* ‘it’s a pity,’ *atid* ‘future = is soon to’).

The complement verb in monoclausal complex predicates is invariably in the infinitive, the most pervasive and multifunctional non-finite form in everyday usage (§2.4 above). Hebrew **infinitives** consist of an initial *lV-* ‘to’ prefixed to a verb stem, varying only in the vowel of the prefixal syllable, which depends on morphophonological factors such as verb template or stem-initial consonant – compare ***li****-gmor* ‘**to**-finish,’ ***le-****daber* ‘**to**-talk,’ ***la****-xšov* ‘**to**-think’ (Berman, 2018c).^7^ And it accords with the general trend in Hebrew for repeating prepositions under coordination (e.g., *hem mityaxasim ba-maamar* ***le****-vaayot polítiyot ve* ***la****-efšarut šel reforma* ‘They refer in the article **to**-political problems and **to.the-**chances of reform’). Structurally, Hebrew modal/aspectual verbs do not undergo processes of grammaticization analogous to forms like English *wanna, hafta, gotta, gonna.* Rather, the prefixal *lV-* infinitive marker is fused, not with the preceding “trigger” but with its associated verb stem in Hebrew, reflecting its status as inflectional rather than clitic.

A second type of monoclausal complex predicate construction takes the form of an inflected trigger verb expressing “lative” aspect, defined as “moving/changing location in order to do something” (Berman and Slobin, 1994, p. 117). These constructions are analogous to what Vasilyeva et al. (2008) term “serial verb constructions” (e.g., *go get it*, *come get it*) in their analysis of the speech of young English-speaking children, coded by them as “one-clause sentences”.^8^ In Hebrew, the triggers in such constructions are restricted to the two basic motion verbs meaning ‘come’ and ‘go,’ invariably in Imperative mood [e.g., *bo(i)* ‘come:IMP.2SG.M(F)’ or *lex* ‘go:IMP.2SG.M’]; and the dependent complement verb may be in the infinitive or in one of two other irrealis mood forms in the language, Future Tense and Imperative Mood (Berman, 2015). These options are illustrated in (6), with (6a) from a university lecturer to his class, (6b) and (6c) from a mother to her 2-year old daughter (in 2nd person feminine), and an aunt to her 2-year-old nephew (in 2nd person masculine).


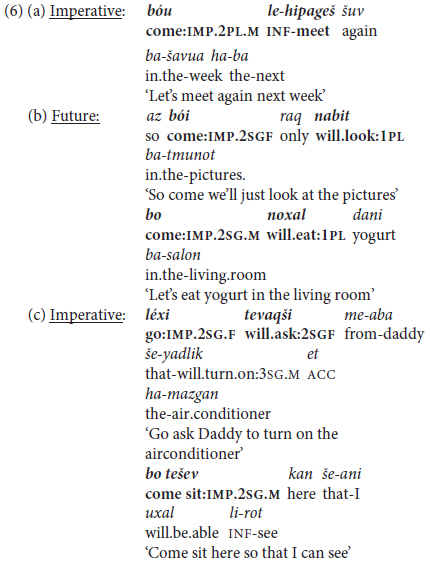


These imperative triggers function pragmatically as “directives” urging the interlocutor to perform an action (if the following verb is in 2nd person) or to cooperate in a given action (if the verb is in 1st person plural, analogously to English *let’s*), so are common in early child language input and output. Unlike “extended predicates” with modal, aspectual, or evaluative trigger verbs, these constructions are confined lexically to two basic lative verbs, and are limited discursively to interactive, typically informal, casual conversational contexts, like the equivalent English “let’s”.

In sum, complex predicates in the form of what are termed “extended predicates” in traditional Hebrew studies were identified as the major instance of monoclausal verb combining in MH, as highly productive and generally applicable across types of discourse. Such constructions manifest relations of bound dependency between the initial modulating element (modal, aspectual, or evaluative) and its associated non-finite complement. They are a key facet of MH grammar, constituting **the** means *par excellence* of elaborating VPs in Hebrew and, as shown below (§5.1), are early acquired and widely used in child as well as adult language.

#### 3.1.2 Mono-Clausal Infinitive Chaining

A major means of elaborating extended predicates is by **chaining infinitives**, as in (constructed) sequences like *hu xašav* ***le-hatxil la-avod*** *šam* ‘he thought **to-begin to-work** there = he contemplated starting to work there,’ *hem nisu* ***le-hamšix la-azor le-tapel*** *bo* ‘they tried **to-continue to-help to-look.after** him = they tried to go on helping to treat him’. In such constructions, the first infinitive functions as both a trigger and a complement element inside more complex and elaborated, verb-phrase constructions. Nonetheless, these are still analyzed as mono-clausal since only the final infinitival element *la-avod* ‘to-work’ or *le-tapel* ‘to-treat’ encodes the conceptual content of the clause, modified by the other, infinitival, elements that precede it. These intra-clausal infinitive chaining constructions meet the three criteria specified earlier for mono-clausal verb chaining, with a high level of inter-verb dependency, since modal, aspectual, and evaluative verbs or verbal operators both semantically and grammatically require a complementary element. These turn out to occur only late in acquisition, rarely with more than two infinitival triggers to a single “main verb” (§5.1).

### 3.2 Inter-Clausal Predicate Chaining

The next category of analysis involves various types of **inter-clausal** chaining of predicates, starting with an intermediate construction that has some but not all of the features of mon-clausal chaining (§3.2.1), followed by bi-clausal and multi-clausal non-finite coordinated (§3.2.2), and finite constructions constructions (§3.2.3).

#### 3.2.1 Intermediate Constructions

Constructions lying between mono-clausal complex predicates and inter-clausal predicate chaining take the surface form of {(NP) V PREP-PRO/N] V-Inf}, representing an intermediate level of Main Clause + Non-finite Complement clauses. On the one hand, like the monoclausal constructions discussed above, the second, complement verb is fully dependent, since its clause cannot stand alone. On the other hand, the subject or topic of the verb of the introductory clause refers to the (typically pronominal) **object** and not the subject of the following infinitive, so that semantically the construction can be interpreted as referring to two distinct situations. This is illustrated by examples from a 2-year-old in (7a) and her mother in (7b), as follows: (7a) **you** will allow/**I** will play, (7b) **Mommy** doesn’t allow/**you** must not touch.


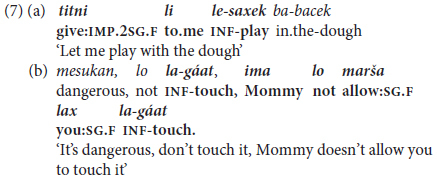


These constructions typically express requests, orders, and prohibitions (e.g., *amar lo la-azov* ‘told (to) him to-leave,’ *bikeš mi-menu la-azov* ‘asked of-him to-leave’ *asar al-av la-azov* ‘forbade on-him to-leave,’ and so differ in semantic content and pragmatic function from monoclausal complex predicates. In Hebrew, they also serve to express causation, corresponding to forms which in English forms may take an unmarked or an infinitival form of the complement verb (e.g., ***let*** *him go* ∼ *allow him to go*; ***mak****e him go* ∼ *force him to go;* ***have*** *him go* ∼ *cause him to go*). In Hebrew, as expected, the complement verb is infinitival rather than a bare stem, although semantically, in both languages, such constructions express similar notions: Permission: *ten li la-léxet* ‘give me = let me to-go,’ *tarše lo la-léxet* ‘allow him to-go’; Prohibition: *asur lexa la-léxet* ‘(it’s) forbidden to.you (= for you) to-go’; Causation: *garam lánu la-léxet* ‘caused us to-go,’ *hevi otánu la-léxet* ‘brought = led us to-go’*-;* Compulsion: *ilec otam la-léxet* ‘compelled them to-go,’ *hixriax otánu la-léxet ‘*forced us to-go’; or Requesting: *bikeš miména la-léxet ‘*asked from her to-go’.

Moreover, in Hebrew these constructions in most cases alternate with **finite** tense-marked subordinate clauses, as in the constructed examples in (8).


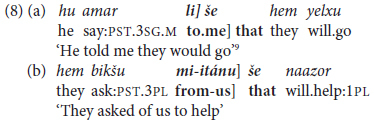
^9^

These constructions, as noted, are typically triggered by *verba dicendi* such as *say, tell, ask* which alternate with non-finite complement clauses, which can also take same-subject reference (e.g., *amar-ti lo] še elex]* ‘told-1SG him] that 1SG’-will.go’ = ‘I told him that I would go’]). In contrast, cognitive verbs (like *know, think, understand*, etc.) tend to require finite complements, and involve a change to a modal or aspectual sense when used in monoclausal constructions. Compare *yodea li-sxot* ‘know (how) to = be able to swim’/*yodea] še hem soxim ‘*knows] that they swim’; *xošev la-azov* ‘thinks to-leave = thinks of leaving, plans to leave’/*xošev*] *še hem azvu* ‘thinks] that they left’. Thus, while semantically, canonical mono-clausal verb chains in Hebrew express modal, aspectual, or evaluative modifications of the main lexical verb, the constructions noted here that employ speech act and mental state-related trigger verbs imply different participants, hence distinct situations. These constructions are mentioned here for purposes of cross-linguistic comparison, but are not noted as a special level of development in the findings delineated in Section “Findings” below.

#### 3.2.2 Inter-Clausal Predicate Chaining by Coordination

Like other Semitic languages, MH makes wide use of *parataxis* as a means of combining parallel or equivalent constructions (see, for Hebrew, Bar-Lev, 1986; Nir and Berman, 2010, and for Arabic, Kaplan, 1966; Johnstone, 1987; Ostler, 1987, 1988). Concern here is with coordination (Haspelmath, 2007) as a means of **inter-clausal syntactic chaining**. Consider, first, coordination of **non-finite** verbs by chaining of two or more “extended predicates” with the basic coordinating conjunction *ve* ‘and’ (Berman, 1996) as illustrated in (9) below.


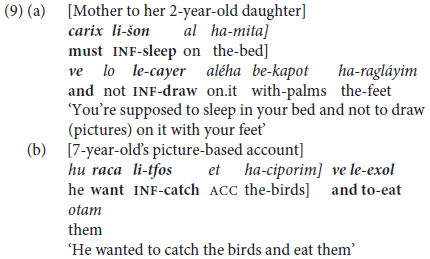


The examples in (10) illustrate more sophisticated and elaborate predicating chains of internally complex clauses in (10a) and of more than two clauses in (10b).


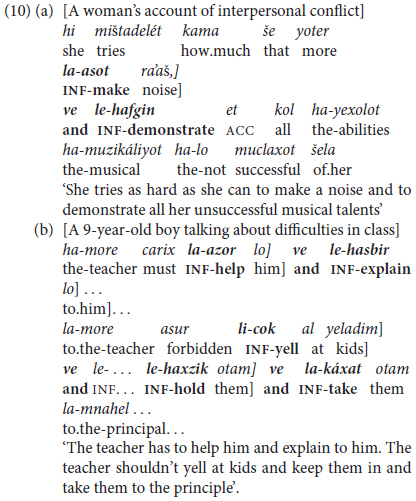


As the examples in (9) and (10) show, unlike their English counterparts, the chaining of infinitives necessarily involves repetition of the bound infinitival marker *le-* or *li-* which is inseparable from its verb stem.

The main type of non-finite **subordinate clause** combining in Hebrew (except for Gerunds which, as noted in §2.4 above, are highly formal and not relevant to child language) occurs with adverbials of purpose. Uniquely to such constructions, the two clauses may but need not be marked by an overt lexical connector, as in (11a) to (11c), from the “frog-story” sample of picturebook narratives, from children aged 5 and 9 years and from an adult respectively.


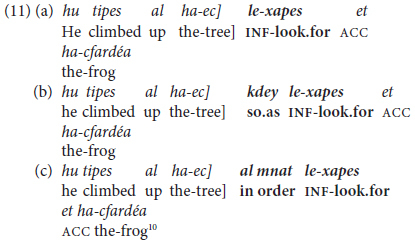
^10^

Another type of bi-clausal predicate chaining is by **finite** coordination. In the present context, this is confined to cases of same-subject deletion in the conjunct clause or clauses. Hebrew (like English and French, but unlike Spanish or Italian) allows pronominalization of the coreferential subject of coordinated clauses, so that subject ellipsis can be viewed here as a cohesive type of clause combining or packaging, as in (12) from two grade-school children.


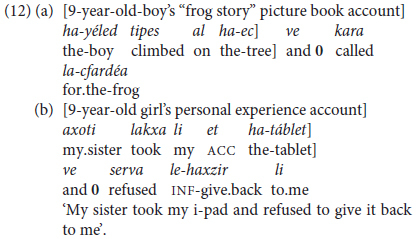


Predicate chaining by means of same-subject coordinate and (one type of adverbial) constructions, both non-finite and finite, is an important feature of MH syntax, one which develops toward late preschool age, as detailed in Section “Inter-clausal Predicate Chaining” below.

### 3.3 Predicate Chaining as a Means of Discourse Connectivity

The final level of verb-predicate chaining considered here occurs in extended discourse, as a facet of what we termed “syntactic packaging” (Berman and Slobin, 1994, pp. 13–15) or “clause packaging” (Berman and Nir-Sagiv, 2009) in earlier analyses of narrative texts. Reference here is to chaining of **chunks** of three or more clauses where only the first makes overt (pronominal or lexical) mention of the topic-subject, and the following are joined by coordination and/or subjectless subordinate clauses. This section is divided between multi-clausal non-finite predicate chaining (§3.3.1) supplemented by chaining of finite coordination in some cases co-occurring with subordinated clauses (§3.3.2). Functionally, these constructions serve the purpose of “topic continuity in discourse” as defined, including examples from Modern Hebrew, by Givón (1983).

#### 3.3.1 Non-finite Coordinated Chains

One kind of discursive chunking takes the form of an initial extended predicate in the form of a tensed trigger *+* infinitival complement going beyond the canonically bi-clausal “extended predicate” construction to include several infinitival predicates marked by overt coordinating conjunctions, as in (10b) above, with more elaborate examples in (13) and (14).


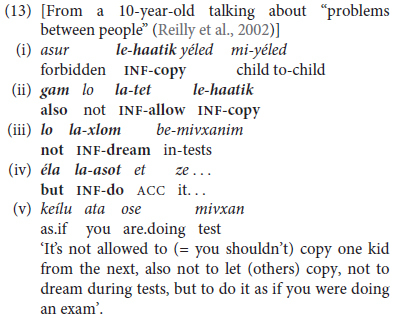


The excerpt in (13) is an extended and varied example of discursive predicate chaining by coordination of five different clauses. These are introduced by a main clause in the form of a present-tense copular clause with a modal *+* infinitival complement in (13-i), with the last clause in the generic present in (13-v). Instead of the repetitive, basic *ve* ‘and’, this boy strings his clauses together either by using the additive particle *gam* ‘also, too, as well’ in (13-iii) and the sophisticated adversative *éla* ‘but’ in the exclusive sense of German *sondern* rather than the basic contrastive conjunction *aval* ‘but’ in (13-iv).

Similar, tightly bound chunks with highly varied chaining of non-finite clauses occurred primarily in adult texts in our sample, as further illustrated in Section “Findings” on Results.

In another, yet more tightly packaged type of non-finite complement (and occasionally also adverbial) predicate chaining, the coordinating conjunction occurs at the beginning and end of the chunk, with **no overt marker** of the relation between the intermediate clauses, indicated by the zero in the example in (14) excerpted from a talk given by a university graduate student, on the topic of interpersonal conflict.


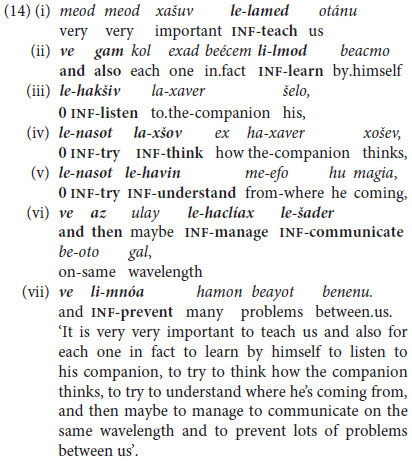


In (14), the speaker chains no fewer than seven coordinated clauses in a single “syntactic package,” marking this relation initially by *ve gam* ‘and also’ in (14ii), and concluding with a clause-initial *ve az* ‘and then,’ followed by *ve* ‘and’ in (14vi) and (14vii). The intermediate clauses are chained without overt marking in (14iii) to (14v), with a repeated clause-internal trigger *le-nasot* ‘to-try to …’ in (14-v) and (14v). This, like other examples from mature speakers in Section 5 below, manifests a high level of inter-clausal dependency at the service of discursive cohesivity.

#### 3.3.2 Finite Clause Combining

Predicate chaining of same-subject **finite** clauses in MH may be seen as lying between coordination and subordination although, as noted earlier, unlike canonical clause chaining languages, Hebrew lacks a third category of non-finite clauses that are neither coordinated with nor subordinate to the finite clause. In terms of the hierarchical schema of different degrees of clause linkage proposed here for MH, the clauses in same-subject finite coordinations are more loosely combined then those in the preceding levels of predicate chaining, since the two (or more) coordinated clauses, like those in (15), can be analyzed as independent of one another syntactically as well as semantically. The examples in (15) are from adult conversational interactions, (15a) with a lexical subject followed by an overt 3rd person pronoun in present tense, the second (15b) with an inflected verb in 1st person past tense.


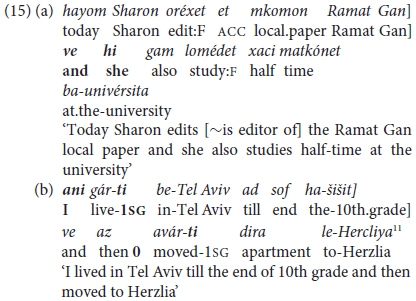
^11^

The excerpt in (16) below illustrates a case of clauses packaged together syntactically that fits more closely into the hierarchy of predicate chaining as defined so far for MH, since it opens with two non-finite correlative coordinated clauses (joined by *o*… *o* ‘or … or’ in the sense of ‘either … or’).


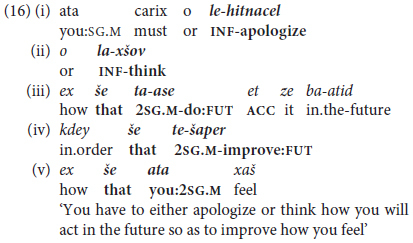


The example in (16) includes five clauses in a single clause package [defined as “a text-embedded unit of two or more clauses connected by abstract linkage relations that are typically but not necessarily identified by overt lexical markers and/or syntactic criteria” (Berman and Nir-Sagiv, 2009); and see, further, footnote 13 below]. The syntactic “package” in (16) contains mixed correlatively coordinated infinitives in (16i) and (16ii) followed by three subordinate clauses with finite verbs, which share generic-reference 2nd person future tense in singular number and masculine gender (Berman, 2005). Given that all the clauses meet the criterion of same-subject ellipsis without an overt pronoun except in the initial clause in (16i) and the concluding clause in (16v), with overt masculine singular *ata* ‘you,’ this string of clauses is analyzed as an instance of predicate chaining in Hebrew, combining both non-finite clauses in (16i) and (16ii) with finite future-tense verbs in (16iii) and (16iv). The fact that the package concludes with what we characterize as a “non-chained” clause with an overt pronoun *ata* ‘you,’ obligatory with the present tense verb in (16v), like 3rd person *hi* ‘she’ in (15a), fails to meet our criterion of “null-subject” dependent clauses, reflecting the asymmetry of verbs obligatorily inflected for person in past and future, compared with non-person inflected present tense in MH, as described in §2.5 above.

#### 3.3.3 Topic Chaining by Juxtaposition

Under this heading, reference is to the most highly fused or integrated type of predicate chaining packages we found in our database for MH: Instances where strings of coordinated clauses are chained together in juxtaposition, without an overt coordinating conjunction like *ve* ‘and,’ *aval* ‘but,’ *o* ‘or’. Consider, first, the personal-experience account of a man asked to tell about an experience he had encountered with violence, in (17): Topic-switching and overt pronouns are marked by underlining. in (17vii) and “clause packages” (Nir-Sagiv, 2008; Berman and Nir-Sagiv, 2009) are indicated by double brackets]] and by a period in the free translation following (17xi).^12^


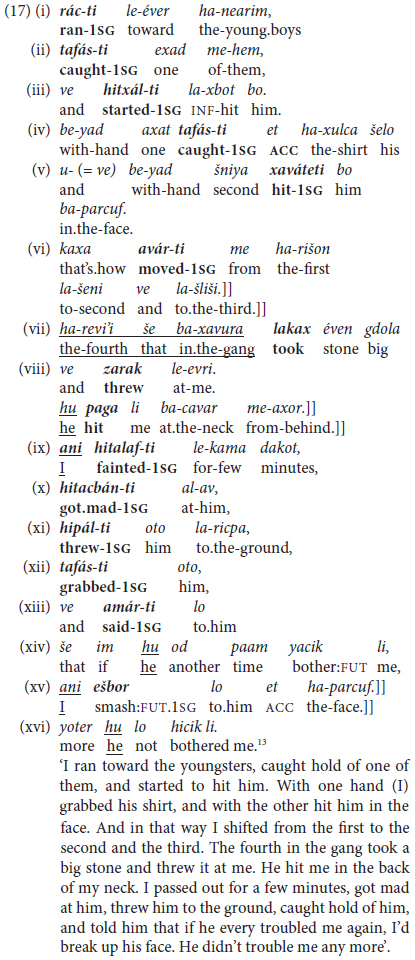
^13^

The text in (17) illustrates chaining of finite person-inflected verbs with or without overt surface pronominal or nominal marking of the grammatical subject, where mention of such a subject typically indicates topic shifting or contrastive emphasis.

An even more extreme instance of **topic chaining** by finite coordination is represented by the last example in (18) below, an excerpt from the picture-book narration elicited from a woman in her 20 s, where the past-tense verbs are marked by the plural suffix *-u* (Berman and Neeman, 1994, p. 324) and a double bracket]] indicates the end of a shared-topic chain. Here, as in general in Hebrew, topic switching is marked by a change in overt subject noun or noun phrase, often but not necessarily by tense-shifting between past and present tense.


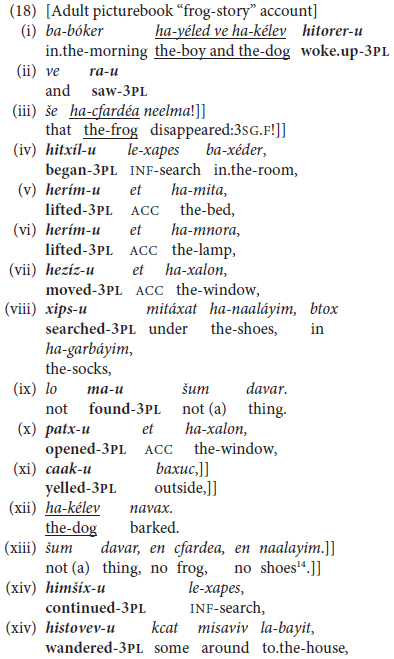
^14^


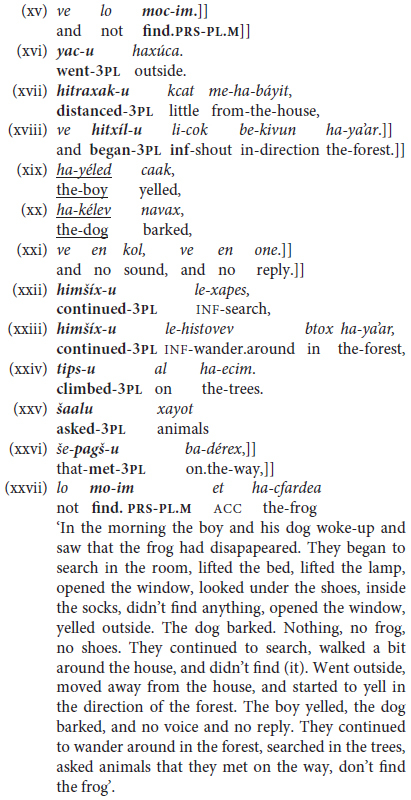


The text in (18) reflects this particular narrator’s personal propensity for use of the rhetorical device of **repetition**, both lexical and grammatical (Berman, 1988). It also reflects other quite general typological features of Hebrew. One is a relatively high-register device for topic-maintenance by the shared past-tense plural suffix switching to a full lexical noun when the topic changes from boy plus dog to boy or dog alone or frog. This reflects an efficient means of indicating switch-reference in Hebrew narrative discourse, even though the language lacks a specific morphological exponent for this purpose.

### 3.4 Predictions

The analytical framework delineated in Sections “Mono-Clausal Complex Predicates” to “Predicate Chaining as a Means of Discourse Connectivity” above, yields the following predictions:

• Development will reflect structural complexity, shifting from early pre-school mono-clauseal non-finite + finite verb constructions (§3.1), via school-age inter-clausal chaining (§3.2), to extended chunks of discourse-motivated topic chaining from adolescence and beyond (§3.3).
• Development at the first level of mono-clausal structural complexity will reveal increased age-related lexico-semantic specification in the types of verbs and verbal operators serving as “triggers,” as follows: modal > aspectual > evaluative.
• The finite triggers in non-finite verb and predicate chaining will increase with age in variety, semantic specificity, and level of usage.
• Development at each level of structural complexity will reveal increased verb/predicate embedding in the number of verbs and/or predicates chained together within as well as between clauses.
• Linkage of finite (coordinated and subordinated) clauses by (optional) deletion rather than pronominalization of the subject/topic shared across the chain will develop late.
• Extended topic chaining, with juxtaposed clauses unmarked by a connective conjunction, will occur only in mature text-construction.

## 4 Database

As detailed in Table 1, the database of spoken Hebrew analyzed in this study consists of two types of corpora: (1) interactive conversations of adults with toddlers (Table 1A) and (2) different types of monologic narrative accounts of children from preschool via school-age and adolescence (Table 1B). On the reasons for reference to “utterances” as the basic units of toddlers’ speech compared with “clauses” for extended narrative texts, see footnote 7 above (and see, further, Dromi and Berman, 1986).

**TABLE 1A:** Sources of adult-child interactive data by elicitation setting, age, size of corpus (total utterances), and reference sources.

Setting	Participants	Age-Range	Size of corpus: # Utterances	References
(i) Adult-child longitudinal	Lior, girl	2.0 – 3.1	12,031	Lustigman and Berman, 2016
	Leor, boy	2.0 – 3.0	13,646	
	Hagar, girl	2.0 – 3.3	8,153	

(ii) Adult-child cross-sectional	20 per year-group	1.0 – 1.11	3,892	Dromi and Berman, 1986
		2.0 – 2.11	8,455	
		3.0 – 3.11	6,752	
		4.0 – 4.11	3,895	

**TABLE 1B:** Narrative database by elicitation setting, number and age of participants, size of corpus (in clauses) and reference sources.

Elicitation setting	Participants	Age-Range	Size of corpus: Total # clauses	References
“Frog-story” narratives”	12 at each	3–4 years	367	Berman and Neeman, 1994
	age-group	4–5 years	451	
		5–6 years	619	
		9–10 years	748	
		20 s – 30 s	734	

“Fight-story” Narratives	12 at each	3.2 – 4.3	124	Berman, 1995
	age-group	5.0 – 5.6	112	
		7.0 – 7.6	118	
		9.0 – 9.11	120	
		20 s – 30 s	422	

Interpersonal conflict narratives	20 at each	Grade IV (9–10)	297	Ravid and Berman, 2006
	age-group	Grade VII (12–13)	502	
		Grade XI (16–17)	390	
		Adults (20 s – 30s)	654	

The first set of data listed in Table 1A consists of longitudinal recordings of three toddlers between 1.6 and 3.0 years in interaction with an adult (Lustigman, 2016a, b). The longitudinal samples are taken from the child language database of the Berman lab at Tel Aviv University, a subset of which is available in the Berman corpus on CHILDES^15^. The children were audio-recorded for a total 1 h per week in their home environment, in everyday interaction with their caregivers. Investigators were university-educated family members (the mother in the case of the two girls, Lior and Hagar, and a paternal aunt in the case of the boy, Leor). These samples provide a richly contextualized data that reflect how relevant verb/predicate chaining constructions are used in everyday speech directed at children, on the one hand, and how and when these constructions emerge in children’s speech, on the other. The longitudinal corpora are supported by reference to cross-sectional data based on single sessions of adult-child interchanges from 80 children, 20 per year-group from ages 1–5 years (Dromi and Berman, 1986).

These interactive data were supplemented, as shown in Table 1B, by extended texts in the form of oral narratives of children between ages 3 years to adolescence, native speakers of Hebrew from middle-class families with no known language or learning disorders, in each case including comparable groups of university educated adults. Sample sizes are given here for these cross-sectional data in terms of total number of clauses per age-group, where a “clause” corresponds to a unified predication, as defined at the outset of Section “Framework of Analysis” above. The narrative corpora were derived from three separate studies, as follows. The first set (i) derives from stories based on the “frogstory” picture book consisting of a booklet of 25 pictures without words depicting the adventures of a boy and his dog in search of their lost frog, which participants were asked to recount while looking at the pictures. Data analyzed here cover 12 children at each of the ages of 3, 4, 5, and 9 years, and a group of 12 college-educated adults. Average number of clauses per age-group ranged from 35 to 46 among the preschoolers aged 3–5, 58–60 at school age 9, and 70 among adults (Berman and Neeman, 1994). The second set (ii) consists of personal-experience narratives of 12 children at each of the ages 3, 5, 7, and 9, and a comparable group of university-educated adults, who were asked to recount to a family member or friend an experience in which they had quarreled or had a fight with someone. This design yielded largely interactive adult-child interchanges among the 3-year-olds as against monologic narrative texts produced by older children and adults. The texts elicited varied greatly in size between children and adults, with number of clauses per text among the two extreme age-groups (3-year-olds yielding 4–21 clauses per session, averaging 13.8 clauses in all and the 12 adult accounts ranging from 13 to 80 clauses, averaging 35.2 clauses across the group, while the older children (kindergarten 5–6 year-olds and school children aged 7–8 and 9–10 years varying far less, averaging between 9.3 and 9.8 clauses per narrative (Berman, 1995, 2001). A third set of narratives (iii) were elicited from 20 adults plus 20 school-going participants at each of three age groups (9–10 years, 12–13 years, and 16–17 years), who were first shown a short wordless video clip demonstrating young people in different situations of conflict – moral, social, and physical – and then asked to tell a story about an incident where they themselves had been involved in interpersonal conflict. In this data-set of narratives, number of clauses per text averaged 15 in the youngest group of gradeschoolers, 25 at middle school, 20 at high school, and 33 among adults (Berman and Nir-Sagiv, 2004, 2009).

This varied database is motivated in principle by the importance of going beyond the early phases of language acquisition noted in the Introduction, including the fact that children’s early complements are confined to particular verb constructions (Diessel, 2004). Thus, in contrast to most psycholinguistic studies relying on semi- or non-structured elicitations of children’s speech, not only do age ranges differ across our sample, but also communicative contexts, including interactive conversations and monologic narratives produced by different elicitation procedures. Moreover, as noted, all the corpora included adult participants as a point of comparison with children’s language.

The following section presents findings from this diverse set of data in terms of different developmental phases, as explained in the Introduction (§1) for different types of verb/predicate chaining as defined and illustrated for MH in the preceding section (§3.1–§3.2).

## 5 Findings

Findings are presented for different developmental phases at each of the three levels of verb/predicate chaining delineated in Section 3, as follows: Mono-clausal verb chaining (§5.1), Inter-clausal predicate chaining (§5.2), and Discursive topic chaining (§5.3). Our predictions were largely confirmed. The general developmental trajectory that emerges is initial usage of bi-verbal mono-clausal chaining, to acquisition of bi- and multi-clause predicate chaining, and on to mature mastery of complex, discursively motivated inter-clausal chunks of predicate chaining.

### 5.1 Monoclausal Chaining

“Complex predicates,” in the form of a finite trigger verb followed by a verb in the infinitive in the same clause, occurred across the data-base (§4), from two-year-olds to adults, in conversational interactions and oral narratives. Consequently, findings for this construction are presented in greater detail than for the constructions described in Sections “Inter-clausal Predicate Chaining” and “Predicate Chaining as a Means of Discourse Connectivity”.

• **Monoclausal Phase I: Isolated Infinitives**

Infinitival forms emerge early in toddlers’ speech (Lustigman, 2012) but, as shown by the examples in (19), from three different toddlers between ages 1.9 and 2.3, they initially serve as isolated predicating elements.^16^


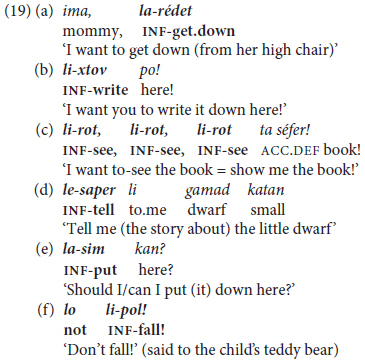


This widespread early use of “lone infinitives” in the form of truncated, rather juvenile constructions reflect various irrealis speech acts, like wishing, requesting, instructing, querying, or prohibiting – not, however, serving reportative or descriptive functions. And they typically follow on earlier emergence of the modal verb roce/roca ‘want.PRES.3SG.M/F’ without any complement. These uses are not characterized here as “pre-grammatical,” since similar uses of lone infinitives occur in adult spoken Hebrew as well, typically but not only from caretakers, parents, and teachers, issuing orders and prohibitions to children (Berman, 2018c). Examples of such constructions in child-directed speech include:

axšav li-šon ‘now (it’s time to go) to-sleep,’ le-exol yafe! ‘to-eat nicely = don’t mess,’ lo li-cok! ‘not to-shout = don’t shout,’ kulam la-šévet ‘all to-sit = everybody sit down!’.

• **Monoclausal Phase II (a): Tensed trigger plus + Infinitive**

Isolated infinitives are soon expanded to mono-clausal verb chaining, as illustrated in (20) recorded from children aged 2.3–2.11 years old.


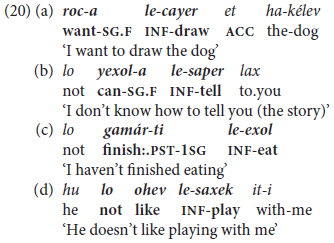


Adult input uses similar trigger verbs in a wider range of inflected forms than their toddlers. These include, for example, *rací-nu* ‘want:PST-1PL = we-wanted,’ *t-uxl-i* ‘FUT.2-can-2.F = you’ll be able to,’ in contrast to their children’s invariant (except for gender-dependent self-reference) *roce* ∼ *roca* ‘(I) want:M~F,’ *yaxol* ∼ *yexol-a* ‘(I) can:M~F’.

In **lexico-semantic terms**, once children start using “extended predicates” in MH, they expand them to a larger set of initiating modal expressions in addition to the initial very widespread ‘want’ in (20a) – mainly *yaxol* ‘can, be able to’ as in (20b) and *carix* ‘must, have to’. These are followed by aspectual triggers like *gamar* ‘finish’ (20c), *hitxil* ‘begin, start,’ followed by an infinitive, and by evaluative expressions such as *ohev* ‘like’ in (20d), extended later to more sophisticated verbs like *maadif* ‘prefer’. Children’s modal expressions are by and large deontic, including permission/prohibition, requests, and judgments rather than epistemic modals referring to hypothetical contingencies, as demonstrated by findings from texts written by school-age children in Hebrew and other languages (Reilly et al., 2002).

Adults’ input to their toddlers, as noted, makes use of a richer repertoire of such expressions, including non-verbal triggers, as in the excerpts in (21) from a mother talking to her 20-month old daughter:


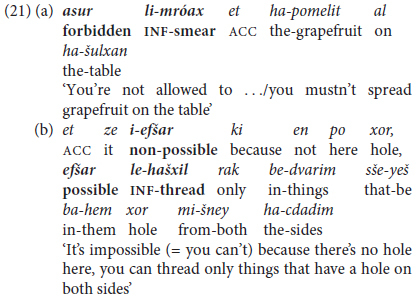

• **Monoclausal Phase II(b): Lative directives**

At the same time as children start using finite triggers with infinitival complements, they also make use of the other type of complex mono-clausal predicate noted in §3.1 above:

directives with the verbs meaning ‘come’ and ‘go’ inflected for tense or mood. The first example of such a construction in (21a) is ungrammatical, since the form *kfoc* is a lone stem without the required infinitival marker *li-* in the form *likfoc* ‘to.jump’.


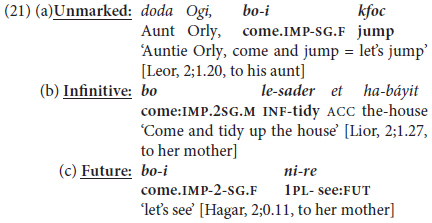


Our findings for use of the canonic “extended predicate” construction illustrated in toddlers’ speech in (19) and in adult input in (20) from the longitudinal sample are compatible with those of Dromi and Berman (1986), as described in Table 1A. Their cross-sectional analysis of adult-child interactions revealed “a gradual rise in the use of more than one verb in the same clause: Around 3% of all clauses at age 2.0–2.11, as against some 6% at age 5 are ‘expanded VPs’ in which modal and aspectual verbs are used together with an infinitival subjectless complement”.

• **Monoclausal Phase III: Later developments in verb chaining**

Two major developments were found beyond early childhood, consolidating in school-age and adolescent usage: (a) **syntactic** chaining of two or more infinitival complements to a single trigger; and (b) elaboration of the **lexical repertoire** of trigger elements inflected for tense or mood.

Chaining of two or more infinitives is grammatically possible up to several such elements preceding the same “head” verb in a single clause (e.g., constructed *hu xašav le-hamšix le-nasot la-azor la-sader*… ‘he thought to-continue to-try to-help to-arrange … = he thought of continuing to try to help arrange …’). These were largely confined to the usage of adolescents and adults. For example, in the occurrences illustrated in (22) through (25), listed by age-schooling level, the first example, in (22a) is largely formulaic.


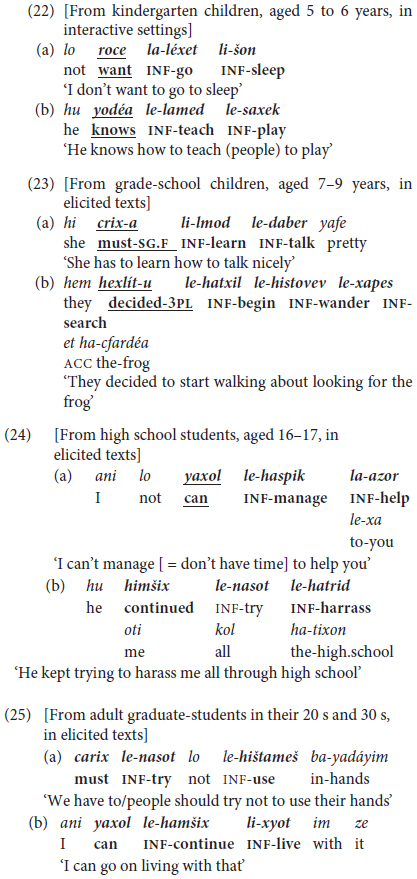


Chaining of more than two infinitives in a single clause, representing what we term Phase III “mastery” of monoclausal complex predicates, is illustrated in (26), from an adult talking about problems with this students.


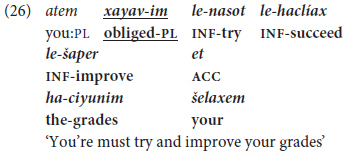


The type of finite trigger verbs also changes in variety and level of usage with age. This is illustrated in (27), from adults’ oral personal-experience narratives and discussions of interpersonal conflict, by semantic class of trigger verb or verbal operator.


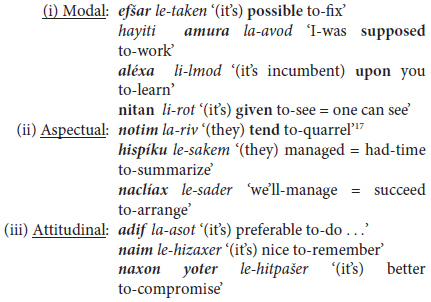
^17^

In sum, mono-clausal complex predicates, typically with a single finite trigger verb, emerge early in development, but they are by no means a purely juvenile phenomenon in MH usage, both spoken and written (see, further, Berman and Nir-Sagiv, 2004). Phase III development reflects syntactic addition of more than a single infinitival complement to a given finite trigger, and lexico-semantic variety and register of trigger elements.

### 5.2 Inter-Clausal Predicate Chaining

As discussed in §3.2, in Hebrew this most typically applies across **coordinated constructions** with the second conjunct clause initiated by the conjunction *ve* ‘and,’ in the form of a null-subject construction, as in (28), repeated from (9) above.


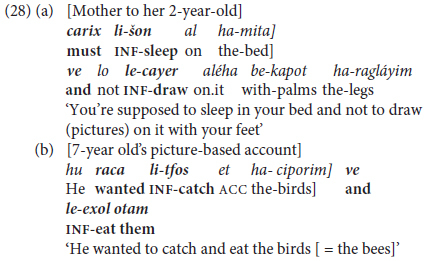


As detailed in §1.2, such constructions are analyzed as instances of **predicate chaining,** since they meet the criteria of (i) expressing two distinct events, hence representing separate clauses, (ii) being non-finite, hence more dependent and less autonomous than clauses with an overt subordinating marker, and (iii) the subject of both clauses being co-referential.

Although adults may use such constructions in addressing their toddlers, such constructions represent a more advanced phase than the monoclausal complex predicates delineated in the preceding section. They did not occur in the corpora analyzed for preschoolers from age 3 to 5 years, while the lone example in the longitudinal simple (*li-kro iton ve le-hadlik or* ‘INF-read newspaper and INF-turn.on light,’ from Leor, 2;7.11), does not really make sense, since typically switchng on a light precedes the act of reading.

Rather, young Hebrew-speaking children opt for different alternatives to non-finite predicate chaining by coordination. These are noted below “Phase I” precursors as to predicate chaining in Hebrew.

• **Interclausal Phase I: Precursors to non-finite predicate chaining:**

This takes three main forms, two in coordinated clauses, and a third mixing coordination and subordination with same-subject ellipsis. The first represents juvenile usage, with repetitive chaining of **finite** clauses introduced by ‘and’ followed by a superfluous and/or ambiguous pronoun or a topic-changing lexical, as in (29), from a picture-book account from a pre-kindergarten child.


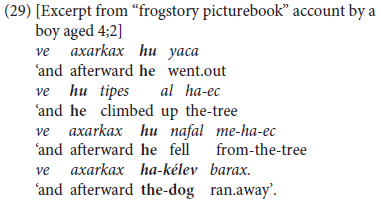


This type of juvenile combining of coordinated clauses is common in Hebrew children’s storytelling, both in the frog-story corpus (Berman and Neeman, 1994, pp. 313–323) and in other narrative as well as interactive contexts (Berman, 1990, 1996; Lustigman and Berman, 2016). We do not analyze this as predicate chaining, since it fails to meet the criterion stipulated earlier of requiring same-subject ellipsis across at least two clauses.

A more advanced type of early clause linkage is confined to **bi-clausal** coordination of **finite** clauses, using same-subject ellipsis as well as lexical and pronominal subjects. This is illustrated in (30) by coordinated constructions common in the narratives of children aged 5–7 years of age.


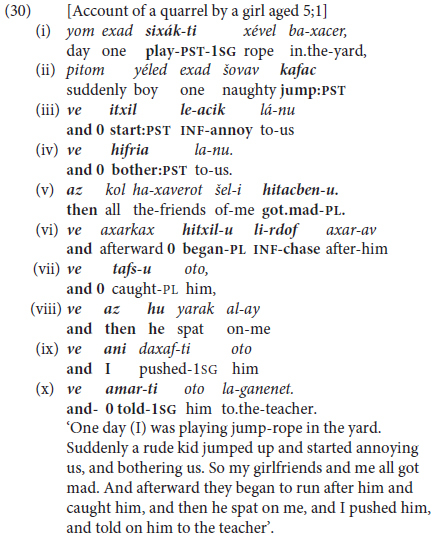


This story represents the “emergence” phase of **initial clause combining** of 5- to 6-year-old narratives in Hebrew. Anchored in past tense, 7 of the 10 clauses open with *ve*, like the more juvenile example in (28), in three instances supported by an overt marker of sequentiality in the form of *az* ‘then, so’ or *axarkax* ‘afterward’. In contrast, lexical subjects or overt pronouns are used to indicate topic-shifting in clauses (ii) and (v) and clauses (viii) and (ix) respectively. **Finite clause combining** occurs with same-subject ellipsis, marked by zero in the gloss, in the rest of the clauses.

A rather more sophisticated alternative in children’s narratives takes the form of **mixing** of finite coordination with subordination, introduced by the invariant subordinating conjunction *še* ‘that’ and also *ki* ‘because,’ as in (31), from a 6-year-old girl.


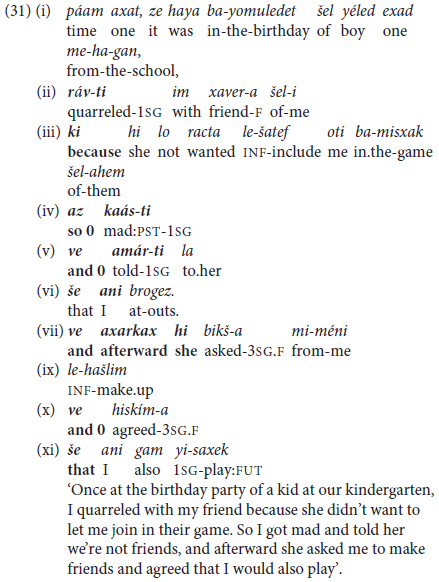


This type of clause combining manifests (Phase III-like) command of grammatical inflection for person and tense, as well as topic-shifting, and coordination interspersed with subordination. However, it does not count as fully mature Phase III topic chaining, since it does not meet the criterion of more inter-dependent *non-finite* predicate chaining stipulated at the outset of §3 above.

• **Interclausal Phase IIa: Bi-clausal chaining of non-finite predicates**

Contrary to what we had expected, bi-clausal predicate chaining occurs only from school-age up, as shown in (32).


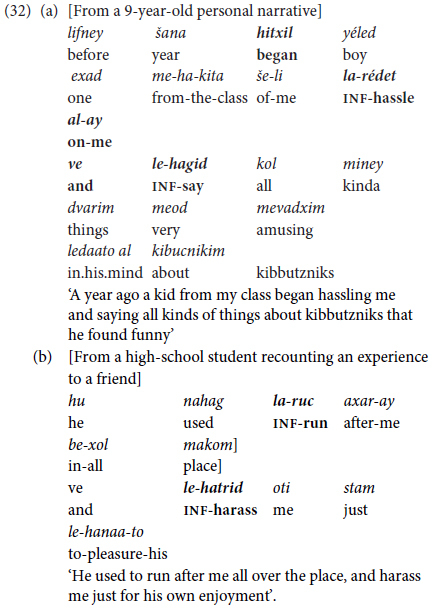


The examples in (32) reflect another important advance in bi-clausal predicate chaining: There is a marked increased in the internal complexity of the clauses that are combined together, with intra-clausal density of information co-occurring with inter-clausal predicate combining.

• **Interclausal Phase IIb: Multi-clausal combining of non-finite predicates**

Predicate chaining in the form of combining more than two infinitival clauses is also a school-age achievement, as illustrated in (33).


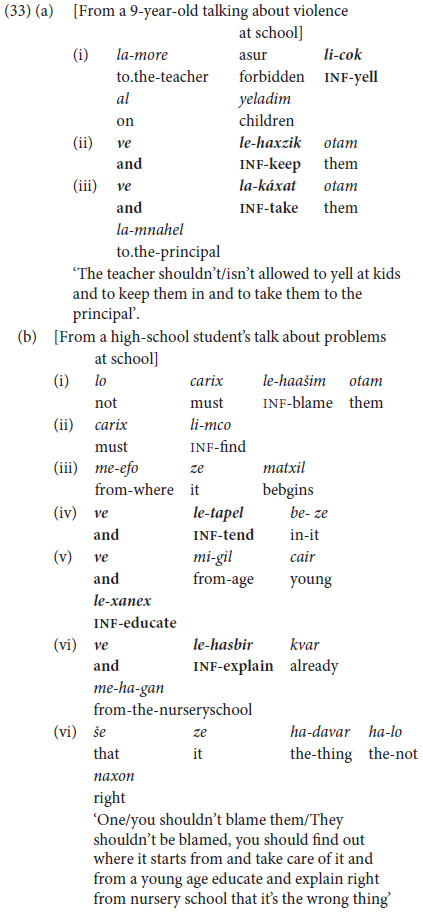


Combining several non-finite clauses with *ve* ‘and’ turned out to be a relatively immature type of construction, en route to what we define as Phase III command of predicate chaining. As shown to some extent in (32b) compared with (32a), more proficient speakers tend **not** to repeat the basic coordinating marker. Rather, they select other options for multi-predicate chaining by alternating different kinds of coordinators with subordination as in (34).


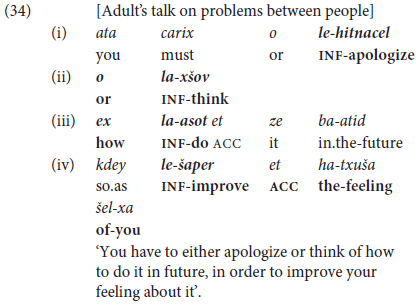


In (33), the speaker starts with a complex type of coordination, expressing alternativeness by the correlative *o*… o ‘either …or’ tightly linking clauses (33i) and (33ii), which are then followed by a question-complement clause in (33iii) and an adverbial of purpose in (33iv). In other words, multi-clausal coordination of the type illustrated in (32) cannot really be defined as having reached “Phase III”. Rather, as shown in the next section, mature multi-clause predicate chaining takes the form of **juxtaposing** of non-finite clauses as a means of cohesive topic maintenance in Hebrew.

### 5.3 Discursive Topic Chaining

A finding we had not expected is alluded to in the preceding paragraph.

• **Phase III of predicate chaining in MH**

This most advanced type of predicate chaining takes the form of packaging together coreferential (typically non-finite) clauses without any overt lexical connector. Such **chaining by juxtaposition** is illustrated by the excerpt from a high-school girl’s narrative in (34), where the auxiliary verb *hayí-nu*.PST-1PL’ is followed by a *participial* form of the verb standing for **habitual past** (as described earlier in §2.3).


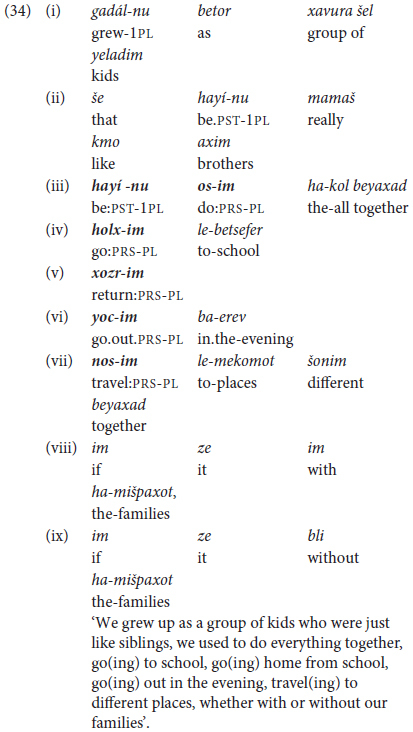


Phase III command of discursive predicate chaining in MH also manifests different choices with respect to the **temporal texture** of a given piece of language. The text in (34) represents a stylistic option for use of a complex predicate to express habitual past tense in current Hebrew, which may but need not be employed when talking about events that used to occur in the past. In contrast, the adult’s picturebook account describing the activities of the boy and his dog in search of their lost frog in (18) above relies on chaining of past tense predicates, interspersed with an occasional present tense verb in clauses (xv) and (xxviii) for highlighting a given situation.

A third option for Phase III advanced predicate chaining is illustrated by the “atemporal” non-narrative text in (35), showing extended juxtapositioning of coordinated clauses. This excerpt, from a college-graduate woman’s talk on problems between people, likewise reveals symmetrical, inflectionally repetitive, chaining, here of infinitives following a noun phrase ‘the nature of man = human nature’.


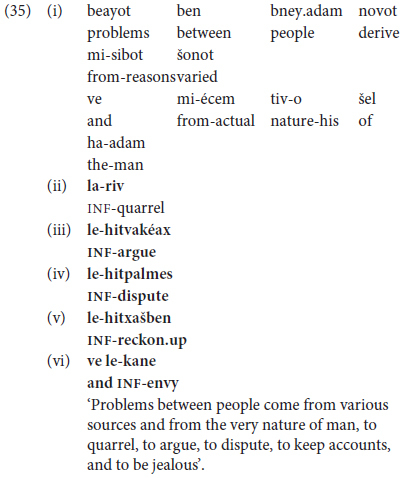


In (35), four infinitives are chained in juxtaposed clauses, headed by the same NP, and concluding with an overt coordinating conjunction.

Such chaining of juxtaposed coordinated clauses, relying on three types of temporality (simple past, habitual past, atemporal present) and interpreted here as representing Stage III in verb/predicate chaining in Hebrew, represent a particularly tight and cohesive or integrative type of clause combining at the service of discursive topic continuity and textual connectivity. Reflecting the highest level of verb/predicate chaining in MH usage, as noted further in the concluding section, these are **rhetorical options** available to proficient users at Stage III in mastery of their language, rather than grammatically required constructions. That is, not only are such extended chunks of discursively motivated predicate chaining confined to mature, highly proficient usage, they represent stylistic choices on the part of individual speakers.

## 6 Discussion

Two main insights emerge from this discussion of verb and predicate chaining in Modern Hebrew. First, the innovative hierarchy in different degrees of verb/predicate linkage, defined here for Hebrew as a non-clause chaining language, mirrors developmental phases in child language. Second, monoclausal chains of finite verbs or verbal operators followed by infinitival complements are grammatically obligatory, and are common from an early age, whereas bi- and multi-clausal predicate chaining represents an optional rhetorical choice on the part of a given speaker-writer in a particular communicative context and are largely confined to more mature language users.

The following discussion touches on the following topics: MH typology, the nature of this study, implications of its findings, and more general significance for child language and cross-linguistic research.

Typologically, the fact that Modern Hebrew lacks canonical types of clause-chaining constructions is attributed to such features as the absence of serial verb and converb constructions in the language (§2). Relatedly, the language lacks simplex verbs with no inflectional marking, so that it has no unequivocal “basic” or neutral verb form; many valence-changing and aspectual categories expressed with auxiliary verbs in other languages are encoded verb-internally by means of the morphological system of Semitic root-and-pattern verb formation. Of particular importance in the current context, the paucity of non-finite constructions outside of more formal registers places a heavy functional burden on infinitives as invariant, minimally inflected forms across the history of the language and in the use of speakers to this day.

Methodologically, the study differs from much work on language acquisition in the nature of its data-base which includes conversational interaction (longitudinal and cross-sectional) between adults and young children as well as different types of monologic discourse (§3). Analysis of findings was conducted in relation to different developmental phases, from initial emergence across structure-bound acquisition and on to discursively motivated usage of the constructions in question. Despite the diversity (in participants, age-groups, and elicitation settings), similar developmental trends were identified across different corpora. For example, in both natural everyday interactions with their caretakers or an investigator as well as in producing personal experience narratives, 3-year-olds were able to use mono-clausal verb chaining with non-finite modals and aspectual verbs followed by finite verbs. In contrast, only adolescents and adults constructed texts using the third and highest level of discursive topic chaining, across different types of narrative settings and elicitation procedures.

This diverse data-base meant that we were able to trace the history of a given construction-type – in this case verb/predicate chaining – from its emergence in early childhood via its establishment as a well-formed grammatical structure, to consolidation as a contextually appropriate discursive device. Another advantage is that a variety of sources makes it possible to conduct comparisons of use of the target construction(s)in relation to different text-types (narrative or expository, descriptive or argumentative) and mediums of expression (written versus spoken). This was not undertaken in the present context, but preliminary analyses suggest that extended chaining of juxtaposed clauses with an overt subject/topic at the outset and an explicit connective conjunction only at the end may be favored in higher-register expository type discourse rather than personal experience narratives. Another suggestion emerging from this study is that the type of elicitation procedure has an effect on use of chaining constructions: These might be particularly favored in narratives based on graphic input in the form of picture-series (Karmiloff-Smith, 1979; Hickmann, 2003) or the “frogstory” picture book used in the present study (Berman and Slobin, 1994), where a series of events is presented graphically to participants – as illustrated above for Hebrew by the juvenile text in (29) compared with the maturely proficient adult text in (18), based on the same storybook. Moreover, alternation in the expression of temporality in verb/predicate chaining may emerge as a function of text-type as well as of target language typology (Kupersmitt, 2015). For example, the chaining of complex predicates in the habitual past with auxiliary ‘be’ plus a participle as in the text in (34) occurs in a narrative account of what the narrator used to do with her friends when she was younger, whereas simple past tense is used throughout in a text like that in (18) from an adult’s picturebook account describing the activities of the boy and his dog in search of their lost frog. And these in turn contrast with reliance on large atemporal present tense in non-narrative contexts as illustrated in the text in (35).

Despite, perhaps thanks to, the typological constraints mitigating against clause chaining in MH, the study enabled us to define an original three-tiered classification as ranging in length (hence, also, structural complexity) from mono-clausal verb chaining (§3.1), to bi-clausal predicate chaining of coreferential, coordinated clauses with same-subject ellipsis (§3.2), and on to discursively motivated chunking of such constructions (§3.3). This hierarchy proved to reflect levels of development, on the one hand, and of syntactic-discursive integration, on the other. The only construction type found to occur across the data-base from toddlers to adults, in all types of elicitation settings, were monoclausal, consisting of a finite trigger verb plus infinitival complement verb. This can be explained both developmentally, as simplex types of verb phrase elaboration, and also typologically, as a key facet of MH syntax in all types of usage. Besides, in contrast to verb chaining in the monclausal complex predicate construction, the two other levels of inter-clausal predicate chaining are not obligatory in Hebrew and can generally be replaced by finite subordinated constructions. Rather, use of predicate chaining, both bi-clausal and even more so in cases of lengthy discursive chunking, represents a rhetorical choice on the part of a given speaker-writer in a particular communicative context (Nir and Berman, 2010).

The fact that speakers of MH were found to rely heavily on paratactic clause combining reflects the favoring of parallel or equivalent constructions noted for Biblical Hebrew (e.g., Hauser, 1980; Berlin, 1985; Polak, 1998; and see, too, references at the beginning of §3.2.2). Unlike the phenomenon of *chiasmus* defined for Biblical poetry as “reverse parallism” or “syntactic inversion,” our database reveals a tendency to use *juxtpositioning* of clauses, each representing a different event or facet of a given event, without an overt lexical connective. This provides speakers of MH with a means of tightly cohesive discursive packaging, as shown with finite-verb clauses in the examples in (17) and (18) and with non-finite clauses in (34) and (35). This, too, reflects a stylistic option favored by some, though by no means all, of the texts in the adolescent and more markedly in the adult corpora.

Another type of alternation between individual rhetorical choices was found in mention or omission of repeated surface subjects typical of Hebrew narrative discourse, representing speakers’ personal preferences for using a pronominal form where this is not grammatically required. In this, Hebrew differs from languages like Spanish or Italian where deletion of coreferential pronouns is obligatory. This is shown by the fact that in an earlier analysis of the cross-linguistic corpus of “frog story” picture-book based narratives, overt subject pronouns are extremely rare in the Spanish texts, they are used by as many as around three-quarters of the English-speaking narrators, and occur in around one-third of all clauses in the Hebrew texts, with little change between texts of preschoolers and adults in this respect (Berman and Slobin, 1994, p. 540). These distributions reflect the grammar of different target languages, here for languages where an overt subject pronoun is or is not required under subject/topic coreference (with English most subject-requiring, Hebrew mixed, and Spanish typically “pro-drop”). And they have not only typological, cross-linguistic, but also developmental significance, since where a feature is deeply entrenched in the grammar of a language, it is typically acquired early, so that its distribution will not change in a given communicative setting (such as picturebook-based narratives) as a function of age/schooling.

In terms of development, as noted, our predictions were largely confirmed. Young children use monoclausal Phase I verb chaining, moving only later to inter-clausal Phase II predicate chaining, while discursive Phase III topic chaining is confined to older speakers.

The study thus extends the notion of developmental phases enunciated in the Introduction to show that within given construction types (here, of verb/predicate chaining), different phases can be defined both within and across over-arching levels of syntactic complexity for a family of constructions in a given language.

The study also reveals the advantages noted by Elman (1993) for “starting small,” with age-related additional length in packaging of speech output reflecting developing cognitive abilities in terms of processing of information, memory load, and pre-planning in production of extended chunks of inter-connected syntactic and thematic content. Moreover, increased inter-clausal length was often accompanied by heavier and more complex information-packing *within* the boundaries of a given clause from middle childhood, as in the example in (31a) from a 4th-grade boy, translated into English as “A year ago, a kid in my class started to make fun of me] and to say all kinds of very things, amusing in his opinion about kibbutzniks]” – the first clause in Hebrew consisting of 9 words (vs. English 14) and the second of 11 (vs. English 14) in the more synthetically inflected Hebrew original. Taken together, these findings for increased chunking within a single syntactic package, combining intra- and inter-clausal density of information, highlight the role of verb/predicate chaining in the concurrent grammatical development of complex syntax and discursive development of information packaging.

The study also underscores another combination of linguistic abilities that develop in tandem: syntax and lexico-semantics. Across the analysis, more complex clause linkage involves more varied, specific, and higher-register use of lexical items such as modals and connectives, as in examples (13) and (25) to (27) above. And it also goes along with higher-level semantic options, as noted earlier for the shift in early childhood from modal to aspectual and evaluative “triggers,” and from deontic to epistemic modal expression.

Numerous open questions remain from this initial study of verb/predicate chaining in Modern Hebrew. For example, cross-linguistic comparisons of parallel or at least corresponding data-bases in different languages might shed further light on typological issues in relation to language development. Detailed distributional analyses of both form and function of such constructions would help to delineate precise developmental trajectories while also shedding further light on the role of communicative context in their use in Hebrew as in other languages – in terms of discursive setting (interactive or monologic), genre (picture-based accounts, personal-experience or fictive narratives, expository or literary prose), and medium of production (spoken or written).

## Data Availability Statement

The datasets generated for this study are available on request to the corresponding author.

## Ethics Statement

Ethical review and approval was not required for the study on human participants in accordance with the local legislation and institutional requirements. Written informed consent to participate in this study was provided by the participants’ legal guardian/next of kin.

## Author Contributions

RB and LL worked in close cooperation with each other and with the editor of this special issue of Frontiers in Psychology throughout the process. RB contributed mainly in the domains of research on the grammar of Modern Hebrew and later language development. LL provided expertise and analyses in the domains of early child language and adult-child interaction.

## Conflict of Interest

The authors declare that the research was conducted in the absence of any commercial or financial relationships that could be construed as a potential conflict of interest.

## Footnotes

1 Hebrew forms are given in broad phonemic transcription, with stressed syllables marked by an accent aigu in the atypical cases of non-final word stress. Coding categories follow the Leipzig conventions of Comrie et al. (2015). Consonantal roots are represented by their abstract historical values, including so-called gutturals like *alef* and *ayin* still represented in the orthography, but not realized in current pronunciation.

2 These details on acquisition are provided here, since they are not considered relevant to the later acquisition of verb chaining detailed in §5.1 below.

3 Since, as noted in the previous section, Hebrew verbs lack a “basic” uninflected form, verbs are listed here in their morphologically least-marked form of past tense, 3rd person, masculine singular.

4 The term “complement” is thus used in the broad sense of Cristofaro (2008) proposal that “there are a number of meanings associated with particular combinations of complement-taking predicates and complementation patterns”.

5 This does not apply in a true clause chaining language like Turkish, which relies on use of converbs for non-finite subordination (Aksu-Koç, 1994, p. 346–347; Berman and Slobin, 1994, pp. 548–552). An analogous situation arises with respect to the relative tightness of packaging represented by same-subject deletion in coordination in languages like English and Hebrew, where it is optional, compared with, say, Italian or Spanish, where it is obligatory (see, further, §3.2 and §3.3) below.

6 Hebrew grammarians treat the ‘extended predicate’ as a mixed bag of constructions, including both (modal) *asuy la-lexet* ‘(is) likely to-go,’ *alul la-lexet* ‘(is) liable to-go,’ *carix la-lexet* ‘must, has to-go,’ *hitkaven la-lexet* ‘meant to-go’, and (aspectual) *omed la-lexet* ‘stands = is about to-go,’ *hitxil la-lexet* ‘start to-go ∼ walk,’ *atid la-lexet* ‘(is) future = due to-go,’ constructed from a tensed verb or modal operator followed by an infinitive as well as the auxiliary *haya* ‘was’ + participle construction noted in §2.3 above (Blau, 1966; Azar, 1977; Cadqa, 1987).

7 As in some other languages, the infinitival marker is the same as the bound preposition meaning ‘to,’ standing for dative case as well as direction toward.

8 We avoid use of the term “sentence” since speakers – let alone young children – connot be credited with a clear sense of what constitutes a “sentence” in their language. Rather, the term “utterance” is used for the unit of young children’s speech output, many of which do not necessarily constitute “clauses” as specified in the present context (see, too, Dromi and Berman, 1986).

9 If the complement clause in (8a) had the same surface form as that of the main clause hu amar li] še-hu yelex ‘he told me] that he would go,’ reference is ambiguous, since the second hu ‘he’ of going could be either coreferential with the hu of telling or refer to two different boys or men. On the other hand, if the subject pronoun is deleted in the subordinate clause hu amar li] še- yelex ‘he told me] that-0 would go,’ the two clauses are necessasrily coreferential (see, further, §3.2.3).

10 Non-finite adverbial subordination in Hebrew typically occurs with a preposition followed by *še ‘*that’ (compare *exarnu* ***biglal*** *ha-géšem* ‘we-were late because-of the-rain’ ∼ *exarnu* ***biglal še*** *yarad géšem* ‘we-were late because came-down rain = it rained’). A few prepositions, however, in addition to those marking purpose as in (11a) to (11c) can precede non-finite subordinate clauses, in which case they are followed by infinitives, not by participles, as in the following example from a high school student: *nisíti le-hasbir la]* ***bli li-fgóa ba*** ‘I tried to-explain to her] without to-offend her = without offending her’.

11 The coreferential verb in the second clause could take an overt subject pronoun *ani* ‘I’ but this is unnecessary both grammatically and referentially, and hence less common in usage (see references in §2.4 above).

12 Topic switches typically indicate the boundaries of what we termed “syntactic packages” (Berman and Slobin, 1994, pp. 538–545), re-defined more explicitly in Berman and Nir-Sagiv (2009) as chunks of texts that meet the following criteria: They (i) contain a syntactically independent “main clause,” to which other clauses in the same package add more information or elaborate on its content and (ii) refer semantically and discursively to the same topic or theme.

13 Marking of past tense verbs for 1^st^ person suffixes distinguishes them from 3^rd^ person verbs which take the stem-form of past tense without an overt marker for person, as noted in §2.5. This also applies to glossing of Example (19) as well.

14 Hebrew present tense existential and copular clauses typically lack an overt lexical verb, justifying classifying (18xiii) as a separate clause.

15 http://childes.psy.cmu.edu/data/Other/Hebre

16 This analysis does not include the “pre-grammatical” phase of uninflected “bare stems” described in §2.1, since they typically occur as lone, hence non-clausal, items.

17 The plural present tense forms of the two aspectual verbs meaning ‘tend’ and ‘manage’ in (27ii) occur without an overt pronoun, despite the general feature of non-person marking on present-tense *benoni* form verbs noted in §2.5. This is because they are used here in generic impersonal, subjectles constructionds (Berman, 2011).
